# Supplementary material for: An intracellular phosphorus-starvation signal activates the PhoB/PhoR two-component system in Salmonella enterica
Source: bioRxiv. 2023 Mar 23:2023.03.23.533958. Preprint. [Version 1] doi: 10.1101/2023.03.23.533958 (PMC10055408; doi:10.1101/2023.03.23.533958)
Supplement: Supplement 3 [file NIHPP2023.03.23.533958v1-supplement-3.pdf]

bioRxiv preprint doi: https://doi.org/10.1101/2023.03.23.533958; this version posted March 23, 2023. The copyright holder for this preprint (which was not certified by peer review) is the author/funder, who has granted bioRxiv a license to display the preprint in perpetuity. It is made available under aCC-BY 4.0 International license.

Supporting Information

Table S1. RNA-Seq summary.

Table S2. PhoB activated genes.

Table S3. Canonical PhoB-regulated genes used as input for MEME software.

Gene name	Strand	Sequence motif identified by MEME	Distance (bp) to translation start site	p-value
phoB	+	**TTTTCAT**AAAT**CTGTCAT**AAAT	-50	N/A
pstS	+<br>+	**TTGTCAT**CAAA**CCGTCAT**AATC<br>**AAGACAT**ATAA**CTGTCAT**CAAT	-86<br>-64	N/A
phoE	-<br>+	**CGGTAAT**AAAA**TGGTCAA**ATTC<br>**TTGTCAT**AAAT**CTTTCAT**TACC	-133<br>-74	N/A
ugpB	+	**AAGTTAT**TTTT**CTGTCAT**TCGA	-72	N/A
phnS	+	**CTTTCAT**CGTT**TTGTCAT**ATAA	-68	N/A
psiE	+	**AATATAG**ATGC**CCGTCAC**ATTT	-44	N/A
ytfK	+	**CTGTAAT**CAAA**AGGTAAA**TATA	-121	N/A
waaH	+	**CTGTAAA**AATT**AATTATG**GCGG	-67	N/A

Table S4. Putative PhoB-motifs predicted by FIMO

Gene name/NCBI locus tag	Strand	Sequence motif identified by FIMO	Distance (bp) to translation start site	p-value
yeaG	-	GTGATATTAAACGGTTACATTG	-341.5	p<0.001
	-	CGGTCACATAACGATCATCAGC	-293.5	p<0.001
	+	TATTCATATGAACGGCTCTTAA	-42.5	p<0.001
yncC	-	CCGTTTTATCTCCGTCATTCCT	-182.5	p<0.001
yiaG	+	TTGTTATTTTATTGTTAATTTT	-281.5	p<0.0001
	+	GTGTAATATTTTTTTAATCTTT	-209.5	p<0.001
phnO	+	CCGCCGTTTAACTGTCATAGAA	-48.5	p<0.001
	-	CCGTCATTATTTATTCTATGAC	-35.5	p<0.001
zitB	-	CCGTTATCAGACTTTTTTTTGC	-98.5	p < 0.0001
	-	TAGACATCATACTGATTTTTCG	-44.5	p < 0.001
katN (*STM14_RS09555*)	-	CAGCCATTTGCATTTCTTCTTT	-184.5	p < 0.001
treZ	-	CTTCCATCTGTCCGTTTTTATC	-325.5	p < 0.0001
	+	TTATCAGCGAACGTTTATTTAA	-207.5	p < 0.001
fbaB	+	ATTTAATAATACCTTTTAAATA	-199.5	p < 0.0001
	-	AAGTGTTAGATCGGTCAAAATT	-165.5	p < 0.001
talA	-	AGGTAACATGACCGTTATAAGT	-130.5	p < 0.001
	-	CCGTTATAAGTTGATAACAAAC	-141.5	p < 0.001

bioRxiv preprint doi: https://doi.org/10.1101/2023.03.23.533958; this version posted March 23, 2023. The copyright holder for this preprint (which was not certified by peer review) is the author/funder, who has granted bioRxiv a license to display the preprint in perpetuity. It is made available under aCC-BY 4.0 International license.

cstA	+	ATGTAAAAAATGGGTAACAATC	-105.5	p < 0.001
ybdD	-	TTGTCACCTGTCATTCAAATGC	-13.5	p < 0.001
STM14_RS03615	-	TAGACATCTAAACGTCTTGATT	-53.5	p < 0.0001
ybdR	+	CTGCTATTTGTCTGGCAATTTT	-152.5	p < 0.001
	+	TGTTAATCAATACTGAATTTAT	-198.5	p < 0.001
wrbA	+	AAGAAATAAATAAGTTATTCTT	-188.5	p < 0.0001
	+	AATAAATAAGTTATTCTTATAT	-184.5	p < 0.0001
	+	CCGTCATAGATTAGACATCATA	-364.5	p < 0.001
narK	+	AAGTTACAAATAATTTAATAAA	-204.5	p < 0.001
	+	ATTCTAAAAAAACTTCAATAAG	-86.5	p < 0.001
STM14_RS12065	-	CCGCAATAATACCGGCATTAAA	-204.5	p < 0.0001
	+	CGGTTATCATTCCTGATTTTTC	-70.5	p < 0.001
STM14_RS15000	+	TGGGCATTTATCCTTAATATCC	-175.5	p < 0.001
	+	CTGACATTACTCGGATATATTC	-151.5	p < 0.001
apeE	+	TTTTAACCAGAATGTTTAAAAC	-82.5	p < 0.001
	-	ATGCCATTCAGCAGTCATCATA	-274.5	p < 0.001
yciG	-	TAGAGATAATATATTCTATATT	-154.5	p < 0.001
yciE	+	TCGAAATAATATTTTTAATTAT	-71.5	p < 0.0001
	-	TTTAAATCAGTTTGTTTTTCCT	-142.5	p < 0.001
bapA	-	TATTTATCTTTATGTTTTATAA	-338.5	p < 0.0001
	-	TTTTAATTTTTTCTTCTTTTTC	-364.5	p < 0.0001
	+	CCTTTATCAATAGATCTTAATT	-241.5	p < 0.001
	-	CAGCAATATTAAATTTTATAAA	-424.5	p < 0.001
	+	ACTTCAAATGATTTTTATAAAA	-437.5	p < 0.001
	-	TAGTCAGAAAAAAATCTTTCTG	-217.5	p < 0.001
phoN2	+	GTATCATCAAACCGTCAACTGC	-81.5	p < 0.001
psiE	-	TGTTTATATTTTGTTCAATAAG	-86.5	p < 0.0001
	+	AATATAGATGCCCGTCACATTT	-55.5	p < 0.0001
	-	CGGGCATCTATATTTTTTATTT	-63.5	p < 0.001
STM14_RS04420	-	AATCTTGCAATAATTCATAAAC	-127.5	p < 0.001
STM14_RS08420	-	TTTACATAATACCATCACATGG	-125.5	p < 0.001
	-	CGGTAATAAATCTTTGGCAATC	-183.5	p < 0.001
	-	AAGATATATTTTTTGAAAACAT	-150.5	p < 0.001
STM14_RS09875	-	CGGACATAAAAATTTATTTATC	-91.5	p < 0.0001
	+	TATACTTAAAACGTTTTTACTG	-27.5	p < 0.001
	-	AATTTATTTATCGCTATTAAGT	-101.5	p < 0.001
STM14_RS19935	+	ACGATCTCAATACGTCTCATTA	-205.5	p < 0.001
STM14_RS23690	-	CATTTATATTAATATCATTAAT	-227.5	p < 0.0001
	+	TATTAATATAAATGATATAAAA	-218.5	p < 0.001
STM14_RS23900	+	AGTTCAGTAATTCGTCGTAATT	-109.5	p < 0.001

bioRxiv preprint doi: https://doi.org/10.1101/2023.03.23.533958; this version posted March 23, 2023. The copyright holder for this preprint (which was not certified by peer review) is the author/funder, who has granted bioRxiv a license to display the preprint in perpetuity. It is made available under aCC-BY 4.0 International license.

Table S5. Bacterial strains and plasmids used in this study

Strain	Relevant characteristics	Source
Escherichia coli		
EC100D	*pir*$^{+}$ (DHFR) host strain used for generation and propagation of plasmid constructs	Epicentre
Klebsiella aerogenes		
ATCC 13048	wild-type	ATCC
***Salmonella enterica* serovar Typhimurium**		
14028s	wild-type	1
EG9054	*phoB*::Km^{R}	2
RB437	*zitB*-HA::Cm (*STM14_RS04415*-HA::Cm^{R})	This study
RB438	*cydB*-HA::Cm (*STM14_RS02420*-HA::Cm^{R})	This study
RB439	*yciG*-HA::Cm (*STM14_RS09540*-HA::Cm^{R})	This study
RB440	*apeE*-HA::Cm (*STM14_RS19030*-HA::Cm^{R})	This study
RB441	*phoN2*-HA::Cm (*STM14_4324*-HA::Cm^{R})	This study
RB443	*phoB*::Km^{R} *zitB*-HA::Cm^{R}	This study
RB444	*phoB*::Km^{R} *cydB*-HA::Cm^{R}	This study
RB445	*phoB*::Km^{R} *yciG*-HA::Cm^{R}	This study
RB446	*phoB*::Km^{R} *apeE*-HA::Cm^{R}	This study
RB447	*phoB*::Km^{R} *phoN2*-HA::Cm^{R}	This study
MP1736	*ΔugpBAEC*::Cm^{R}	This study
MP1737	*ΔglpT::*Gm^{R}	This study
MP1738	*ΔuhpT::*Ap^{R}	This study
MP1739	*ΔpgtP::*Tn*10* (Tet^{R})	This study
MP1778	*ΔushA*::Km^{R} (*STM14_ RS00735*::Km^{R})	This study
MP1779	*ΔushA2*::Gm^{R} (*STM14_ RS03085*::Gm^{R})	This study
MP1780	*ΔushA3*::Tn*10* (*STM14_ RS21590* ::Tn*10*)	This study
MP1784	*ΔphnWRSTUV*::Cm^{R}	This study
MP1785	*ΔaphA*::Cm^{R}	This study
MP1796	*ΔushA*::Km^{R} *ΔushA2*::Gm^{R} *ΔushA3*::Tn*10* (Tet^{R}) *ΔaphA*::Cm^{R}	This study
MP1251	*ΔpitA::*Ap^{R}	3
MP1252	*ΔyjbB::*Km^{R}	3
EG6537	*argH::*Tn*10*	This study
MP50	*ΔmanA::*Cm^{R}	4
MP2133	Km^{R}-*tetRA-ugpBAECQ*	This study
MP2134	Km^{R}-*tetRA-ugpBAECQ* *ΔglpT::* Gm^{R}	This study
**Plasmids**		
pSIM6	rep$_{pSC101}$^{ts} Amp^{R} P$_{CI857}$-γβexo	5
pKD3	rep$_{R6K\gamma}$ Amp^{R} FRT Cm^{R} FRT	6
pKD4	rep$_{R6K\gamma}$ Amp^{R} FRT Km^{R} FRT	6
pKD4-Ap^{R}	rep$_{R6K\gamma}$ Amp^{R} FRT Ap^{R} FRT	4
pKD4-Gm^{R}	rep$_{R6K\gamma}$ Amp^{R} FRT Gm^{R} FRT	4
pKD4-Tn*10*	rep$_{R6K\gamma}$ Amp^{R} FRT Tn*10* (Tet^{R}) FRT	4
pBbB2K-GFP	rep$_{pBBR1}$ Km^{R} *tetRA-gfp*	7
pGFP	rep$_{p15A}$ Cm^{R} promoterless *gfp* vector control	2
pP*pstS-gfp*	rep$_{p15A}$ Cm^{R} P*pstS-gfp*	2

bioRxiv preprint doi: https://doi.org/10.1101/2023.03.23.533958; this version posted March 23, 2023. The copyright holder for this preprint (which was not certified by peer review) is the author/funder, who has granted bioRxiv a license to display the preprint in perpetuity. It is made available under aCC-BY 4.0 International license.

pGFP$_{AAV}$	rep$_{pMB1}$ Amp^R promoterless *gfp*$_{AAV}$ vector control	2
pP*phoB*-*gfp*$_{AAV}$	rep$_{pMB1}$ Amp^R P*phoB*-*gfp*$_{AAV}$	2
pP*pstS*-*gfp*$_{AAV}$	rep$_{pMB1}$ Amp^R P*pstS*-*gfp*$_{AAV}$	2
pFPV25	rep$_{pMB1}$ Amp^R promoterless *gfp* vector control	8
pP*pstS*-*gfp*	rep$_{pMB1}$ Amp^R P*pstS*-*gfp*	2
pUHE-21–2-*lacI^q*	rep$_{pMB1}$ lacI^q Amp^R; vector control	9
pUHE-YjbB	rep$_{pMB1}$ lacI^q Amp^R P*lac*-*yjbB*	This work
pUHE-PitA	rep$_{pMB1}$ lacI^q Amp^R P*lac*-*pitA*	This work
pUHE-Pho89	rep$_{pMB1}$ lacI^q Amp^R P*lac*-*pho89*	This work

bioRxiv preprint doi: https://doi.org/10.1101/2023.03.23.533958; this version posted March 23, 2023. The copyright holder for this preprint (which was not certified by peer review) is the author/funder, who has granted bioRxiv a license to display the preprint in perpetuity. It is made available under aCC-BY 4.0 International license.

Table S6. Oligonucleotides sequences used in this study

Name	Sequence (5'→ 3')	Purpose
801	ACGCGTGATATCGCGCATC	*ugpBAEC*::Cm^R verification
802	CACAAAAAGAGAGATAACCGATGATATCGTTACGAC ATACAGCTTCATATGAATATCCTCCTTA	*ugpBAEC* inactivation
803	TGTTGCAGCAGGACAGCGGGACGCCGCAGCCTGACA TCCCGCGTGTAGGCTGGAGCTGCTTC	*ugpBAEC* inactivation
782	AGCGCGCTTGCCAGCGGCG	*glpT*::Gm^R verification
783	CGCTGGCAGATTTTCCTGGGGATATTCTTTGGCTATG CCGGTGTAGGCTGGAGCTGCTTC	*glpT* inactivation
784	CGCTTGCAGCGACAGAACCGCCCAGGTAACCAAACA GACCATATGAATATCCTCCTTA	*glpT* inactivation
779	ACCAGGTGCGCAAGCCGAC	*uhpT*::Ap^R verification
780	GCGGCGTAAAATGTGGTTCAAGCCGTTCATGCAGTCC TGTGTAGGCTGGAGCTGCTTC	*uhpT* inactivation
781	ATAAGCGAAGGTGCCCTTGATACCGTCCGCAGCGCCG ATGCATATGAATATCCTCCTTA	*uhpT* inactivation
776	AGGGCAATCGGCGCATAAA	*pgtP*::Tn*10*
777	CCGGAAAAAGTCCAGGCCACATATGGTCGATATCGT ATACGTGTAGGCTGGAGCTGCTTC	*pgtP* inactivation
778	GCCAGCCTGCCAGTAGCGTGGAGGGAATCGCCGCCC ACTCCATATGAATATCCTCCTTA	*pgtP* inactivation
898	ATTACCTGATCGGCTTCGA	*ushA*::Km^R *verification*
899	CGACTACAGGCATTGTTTTTCTTTAATGTAGCGTAAA TGGGTGTAGGCTGGAGCTGCTTC	*ushA (STM14_ RS00735)* inactivation
900	GTATGTTATGCGGCCGGTTATCGACCGCATAACATTA TGGCCATATGAATATCCTCCTTA	*ushA (STM14_ RS00735)* inactivation
901	ATGCGATGTTGGAGATAGT	*ushA2*::Gm^R verification
902	AGGTAATTTCTGCGGTTGATATTGAGTCAGGGAGAGA AAGGTGTAGGCTGGAGCTGCTTC	*ushA2* (*STM14_ RS03085*) inactivation
903	TAAGGTTGCGCGCCATCAGGCAGAAATGGCTATCCGT ACCCATATGAATATCCTCCTTA	*ushA2* (*STM14_ RS03085)* inactivation
904	GCAGGCATTAATGGTGAACA	*ushA3*::Tn*10* verification
905	TAACGTAGCGAATCTTTATATGACTGAAAGGGACTTA TTTGTGTAGGCTGGAGCTGCTTC	*ushA3* (*STM14_RS21590*) inactivation
906	CATGTCACGCCGCGACACTGAACGCGCCGCGGCAGG GGAAACATATGAATATCCTCCTTA	*ushA3* (*STM14_RS21590*) inactivation

bioRxiv preprint doi: https://doi.org/10.1101/2023.03.23.533958; this version posted March 23, 2023. The copyright holder for this preprint (which was not certified by peer review) is the author/funder, who has granted bioRxiv a license to display the preprint in perpetuity. It is made available under aCC-BY 4.0 International license.

895	GCGTTATGGTCAGATAGT	*aphA*::Cm^R verification
896	ATCTTAATAATTATAATATTTTGAATTTTAAGGGAAA ACCCATATGAATATCCTCCTTA	*aphA* inactivation
897	AAATCATGCAAAAAAGGAGAGCCTGTCGCTCTCCTG ATTTGTGTAGGCTGGAGCTGCTTC	*aphA* inactivation
916	GCTCTATCTGCGCGAGC	*phnWRSTUV::*Km^R verification
917	GGGTTTGCCAATGTGAAGGTGTATCGTCCGTAATTCC TTTGTGTAGGCTGGAGCTGCTTC	*phnWRSTUV* inactivation
918	AGGCCGAATAAGCGACAGCGCCATCCGGCAGTTATTT TACCATATGAATATCCTCCTTA	*phnWRSTUV* inactivation
1688	GGCGCGGACGTGTTACTTAA	*yhjY-HA*::Cm^R verification
1689	AGCGATTATTTGTATACCTTGGGGGTGAGCGCCAGGT TTTATCCGTATGATGTTCCTGATTATGCTTAGCATATG AATATCCTCCTTA	*yhjY-HA*::Cm^R tagging
1690	AGCTGTTATCACTGCGTTTCGATTATAATTTTTAAGTT AGTGTAGGCTGGAGCTGCTTC	*yhjY-HA*::Cm^R tagging
1691	GCTACGATTCAGATGGAGTA	*zitB-HA*::Cm^R verification
1692	TCATCTGAATCAGACATCGTCCGGGCATGTTCATCAC CATATCCGTATGATGTTCCTGATTATGCTTAGCATATG AATATCCTCCTTA	*zitB-HA*::Cm^R tagging
1693	AAACAGCGCGCGGGAGCGAGGATCGCGCGCGCTTTC CCGTTAAGTGTAGGCTGGAGCTGCTTC	*zitB-HA*::Cm^R tagging
1694	GCCGAATATTATTCCACCGG	*cydB-HA*::Cm^R verification
1695	CGTCTTCCGTGGAAAAGTGCGACATGGTGAGGGATAT CACTATCCGTATGATGTTCCTGATTATGCTTAGCATAT GAATATCCTCCTTA	*cydB-HA*::Cm^R tagging
1696	ATACGACTCGCCGCGACCATTTTTTCACTTTAACCATC AGTGTAGGCTGGAGCTGCTTC	*cydB-HA*::Cm^R tagging
1697	AACATCGTGGTGGTTCAGG	*yciG-HA*::Cm^R verification
1698	GGTCAGAATAGTCACGGCGGACGTAAATCCGATAAT TCCTATCCGTATGATGTTCCTGATTATGCTTAGCATAT GAATATCCTCCTTA	*yciG-HA*::Cm^R tagging
1699	TGCTTGATAAAAGCATGTGTTATATTTACATTACAGT AAATCGTGTAGGCTGGAGCTGCTTC	*yciG-HA*::Cm^R tagging
1700	ACTGGGTTGATATCGCGAT	*apeE-HA*::Cm^R verification
1701	CAATCAAACCCGTTATAACGTTGGGTTTAGCGCCCGA TTTTATCCGTATGATGTTCCTGATTATGCTTAGCATAT GAATATCCTCCTTA	*apeE-HA*::Cm^R tagging
1702	CACGAACGAACGGGGTTGGCCCTCCCTGGCGTGTCAT CAGTGTAGGCTGGAGCTGCTTC	*apeE-HA*::Cm^R tagging
1703	TGCTACAGACAGCGCTGCCG	*phoN2-HA*::Cm^R verification
1704	GATCTGTCCGCTGCTTACGAGATGGCGAGAAAAACG CGCTATCCGTATGATGTTCCTGATTATGCTTAGCATAT GAATATCCTCCTTA	*phoN2-HA*::Cm^R tagging

bioRxiv preprint doi: https://doi.org/10.1101/2023.03.23.533958; this version posted March 23, 2023. The copyright holder for this preprint (which was not certified by peer review) is the author/funder, who has granted bioRxiv a license to display the preprint in perpetuity. It is made available under aCC-BY 4.0 International license.

1705	CATCCCGGCTTACGTTTTATAAGCCGGGTGACAACGT CAGTGTAGGCTGGAGCTGCTTC	*phoN2-HA*::Cm^R tagging
2075	GGCGGCTTACGCTGGAGCAAATTGCCGTTCGCTTACG TGCCTGGGCACTAGTGCTTGGAT	Generation of *km^R-tetRA- ugpBAECQ*
2076	ACTCAGCGCCAGTCCTAAAGCTGTATGTCGTAACGAT ATCATATGTATATCTCCTTCTTA	Generation of *km^R-tetRA- ugpBAECQ*
2077	CCTTCGATTCCGACCTCA	*km^R-tetRA-ugpBAECQ* verification
2078	GCGCTCAGGTTCTGTTCA	*km^R-tetRA-ugpBAECQ* verification
2079	CGCATCGCCTTCTATCGC	*km^R-tetRA-ugpBAECQ* verification
2080	ACTCGCTGCGAATCCGGAT	*km^R-tetRA-ugpBAECQ* verification
W3392	TATCGCAGGATCCACGTATAACGATAAGGAGAAC	*yjbB* cloning into pUHE-21–2-*lacI*^{q}
W3393	TATCGCACTGCAGATGACGGGCCTACGCGCATG	*yjbB* cloning into pUHE-21–2-*lacI*^{q}
W3394	TATCGCAGGATCCGATAATGCGCCGCGTTCATG	*pitA* cloning into pUHE-21–2-*lacI*^{q}
W3395	TATCGCACTGCAGTTAAATAATCTTCAGGGAA	*pitA* cloning into pUHE-21–2-*lacI*^{q}
W3831	TTAGCCCTCTGGCAAATCGTTTTCTAAGGACTTTAAG AAGGAGATATACATATGGCTTTACATCAATTTGA	*pho89* cloning into pUHE-21–2-*lacI*^{q}
W3832	GATCTATCAACAGGAGTCCAAGCTCAGCTAATTAAGT TATGTCATTTGGTATTCC	*pho89* cloning into pUHE-21–2-*lacI*^{q}
146	AATCCAGATGGAGTTCTGAGG	pUHE-21–2-*lacI*^{q} sequencing
153	GTCTCATGAGCGGATACATAT	pUHE-21–2-*lacI*^{q} sequencing

bioRxiv preprint doi: https://doi.org/10.1101/2023.03.23.533958; this version posted March 23, 2023. The copyright holder for this preprint (which was not certified by peer review) is the author/funder, who has granted bioRxiv a license to display the preprint in perpetuity. It is made available under aCC-BY 4.0 International license.

Cis regulatory elements of PhoB-regulated genes

Putative promoter(s) were predicted with SAPPHIRE (10).

Putative Pho boxes on the **positive** and **negative** strands were predicted with MEME Suit (11).

Primary and **secondary** transcription start sites have been experimentally inferred (12, 13).

Putative translation start/stop sites were obtained from the genome annotation (14).

Canonical PhoB-regulated genes

>*phoBR*

gccagcgacaataatggcatccacctgatgggcctgcgcggtctccagcagccagtccagaaaagcctga
tgctccgcggcgcggcttttactgtagaaatttgtcccagatgccagtcagaggtgtggaggatgcgca
taattgttccatgcaaaaaaagcgtgaacgggattatacacgtcatcccttccattttgggcgcaattt
accgccggtacacggtaatgcatggtttcaccggtgtcataaatcatcaacatgctgtcaatgccgcctt
tttttttcataaatctgtcataaatctgacgcataatggcgcgGcattgataactaacgactaacagggc
aaatt**ATG**

>*pstSCAB-phoU*

ggtcggtggcgcaatcgccgggggactgtccatgtatttcggttgtacactgatggcgccacac
ggtggcctctttgtcctggcgatcccgcatgcggtagaacatgtgatgcaatatctgctctcga
ttgccctgggcacgattgtctgcggcctgatgtacgcgctgttgaaaccgtctgcggttgcgca
aacagtctaattcatctccccctgcaggctggtctggtgtctccccgggccagcttttttttatt
tccattgtcatcaaaccgtcataatcaagacatataactgtcatcAatttgtcctattttgctc
atcGtagcaactcaaacaacgatttaccgaaaccgtgcaggagacatt**ATG**

>*phoE*_unknownTSS

ccgttcaaaataagaaataaagaccaaatgaacgttttagcaggactggctccggttgccaaca
acctgtacgcgtagcgtgaaattttgttgcgcaggatcagcaagcgtagcggcggaatttgacc
attttattaccgcaacaattaaacatatttttttaaaaaaattctcactttgtcataaatcttt
cattaccgaacgttaaaaaccttcctgtttttttaccgggtttcccgacaaatcatagcgcgtaa
ttaaaacaggaatggaa**ATG**

>*ugpB*

aatcccggcgacgctccgcctggttgggttacggcatgtgcgccgcccccgagaataatgtcgt
acctatccgtgccgtagctcccactttttctttttccggcgctttcgctgccttgccatctctctg
tcgccttactatctttttttttGtaataaaaaagttatttttctgtcattcgagcatgtcatgtt
acccccgcgaacataaaacgcgtgatatcgcgcatcccggcacaaaagagagataaccg**ATG**a
tatcgttacgacAtacagct**TTA**

>*phnS*_unknownTSS

ggatgcgccggatgttggctgcgggacttatccggcctaccaatcgtatcgtctcacggctatc
cggataagcgcagcatcatccggcgtggactgatttatttctctttcatcgttttgtcatataa
gccgtttagcgtaaaagcaaacctggtataggccagaaaatgcaacgtacgctatgaggctat
tacg**ATG**

>*psiE*

aggcgtatatgtttccgtgccgctggatatcttctcgtccggcccaacccgaagtcgtgcggca
attggctggacgccgctgacgcgtgacggtggtcaacagcttggccgtaagtttggcctgtatg
atatgaccagtgacaggagcgtaaatttccgttaatactcaggccggataaggcgtttacggcg
ctatccggcaaattatcttattgaacaaaatataaacataaataaaaaatatagatgcccgtca
cattttgtgcgttatacagaaaccTcgccgcagagaaagagggggctgtt**ATG**

bioRxiv preprint doi: https://doi.org/10.1101/2023.03.23.533958; this version posted March 23, 2023. The copyright holder for this preprint (which was not certified by peer review) is the author/funder, who has granted bioRxiv a license to display the preprint in perpetuity. It is made available under aCC-BY 4.0 International license.

>*ytfK* (STM14_RS23110)
attccttctttattttttgcaggtgatccgaccactttgggccgatagttaatcatatgtgcg
attgatgctttttcccgcaaaggggatgccagtttgcgggcgggcgcacacttcctgtgaaaaa
tgaaggcatatactgagAaaaatgagctgatgtttagataattctgaataactgtaatcaaaag
gtaaatatacttatgcacActggaaacgacgtagatatggtctatagtcatatgGcattaaaat
ttgcgccttaaaactgttgggccgattgtggcatcgcaagggcgtaatactctgcaggagacaa
ca**ATG**

>*waaH*_unknownTSS (STM14_RS19605)
gtgtcattcgccaacctttttttgttagggaaaatctggaaagccgtaaagaattgtcatagaca
tcaagcattcgtaattgcgctttactcttatttactcgctaacgtcacgctctactctgagtt
ttgtgcttgcttttttactgtaaaaattaattatggcggcttaatagtttcttaatagagccaca
gtataaaggcagggtaaattaaggtttttctggtaatcgtt**ATG**

Putative PhoB-regulated genes

>*yiaG* (STM14_RS19310)
ttatctgcatcaatgcttaatttgttatttattgttaattttgacctgacactgcgataaaa
aacgcggcaagaaagctttccagcggggagtgtaatatttttaatctttacaattattttct
gaaagacggatataccttcctggatcaaattgttagcgtcctaaaagtcgcgtagcgtaaggca
tcgaaacgaatcgatagcgcttcctggcggcgcgtcgcccccccacccgcaagtttcccgactat
tcttaagagGcttcgatgcatttcacgatcccgctgtgtgatttacaggagttctca**ATG**

>*yeaG*
ccataattccttattcaaatgttctgcggcaaaagtgtaaccgccaatacctgtaagcccaact
attttacacaatgtaaccgtttaatatcaccacaaccgattattttcagatgatcggctgatga
tcgttatgtgaccgggaacgcttttctgatatccaccagccttttctacctgatgagttattg
atatgtcatcgaaatccactgacgcgtacaggcaagtttgcaaatgccatctacgcttaatgt
tAagaaggtgtatcaccggacacgttaatcttctgaccaataaaatggcatgagagttgctttt
tttttcctctagcagagacggcgttcagtctacctcttccgggagcctctactattcatatgaac
ggctcttaacatgtgcgaaaaaacgaaaggatggcatatc**ATG**

>*yncC*
aatggcggcattatgtcgccataaaaatgtgcaaattttaaaattgcggctttacttatattcA
tcattcagatcgcggtggggcagcttatctccctcagcggcctgaaccggtcaaaaatctcgat
acatgaaggaatgacggagataaaacggttgccataaagaaacctgtattgttgtaaaagatga
gaatatcacgcaagtaaattatcactttatttatttggccgttcaggcctggattatcttttt
tacaattaagttccgaaacttcttcgggactcgattgcgatgttgttctgccaagaggtgaagg
aaggaaa**ATG**

>*phnO*
gctttacgctggtgcttgatacccaggatgtagcagaagggaagcgctggtttgataaccttgc
cgcacaagggcaaatcgaaatggactggcaggagaccttctgggcgcagggtttcggcaaggtc
agcgatcgtttcggtgtgccgtggatgattaacgtggttaaacatcagcctgccacctaactcc
ccgggaggctcgccctccccttgccatcagagtgatctttggctgcacgttatccgccgttta
actgtcatagaataaataatgacggtaGtttgctacagtctggcgaaag**ATG**

>*ybgS* (STM14_RS04420)
tgtttattccagtgttgccgtacttatcccaatgaatgcaatccctccaatctatctcttcaat
taaatagtgtaaacgggcttttacactttgaacggaataatcctggaattcaggaaaaaaacgc
acaaatgtagcgaaaaatgggatctaatctacacttttaactGtaaccactctgtttatgaat
tattgcaagattctctgctgcgttaacccgcggcggcgaacgcttttatcccttatttgagga
tttgactgacacgtgcactgttggaagaggttatccgacatatccaccataacaggagcatctt
ATG

bioRxiv preprint doi: https://doi.org/10.1101/2023.03.23.533958; this version posted March 23, 2023. The copyright holder for this preprint (which was not certified by peer review) is the author/funder, who has granted bioRxiv a license to display the preprint in perpetuity. It is made available under aCC-BY 4.0 International license.

>*zitB*

cgacatcaataccaacggcaatactaacagcacgatgcagcatccagacggttcaaccatgaat
catgacggaatgaccaaggatgaagagcataaaaataccatgtgtaaagacggtcgctgcccgg
atattaataaaaaagtggaaaccggtaatggcgtcaataatgacgtgaataccaaaaccgacgg
taccacacagtaatgcaaaaaaaagtctgataacgggagagctttcgctctcctttttattttg
tcagcgaaaatcagtatgatgtctacagtattgatGagaataacaaaggaatgacgtt**ATG**

>*yciE_katN*_unknownTSS

ggagaaaaacgaagtacgcgatgcagcgcttatcgccgcggcgcaaaaagtcgagcattacgaa
atcgccagctacggcacgctagccaccctggccgagcagctcggctatagcaaagcattaaaac
tgctcaaagaaaccctcgacgaggaaaaacaaactgatttaaaacttaccgatttagcagtcag
caatgttaataaaagtgctgaacgcaaatcgaaataatattttaattatcagcttgcatgatt
ccgattttattatcggagcagattatcacgcattgaggaatgtaaa**ATG**AATTATACTGAACAT
TATCATGACTGGCTTCGTGACGCCCATGCCATGGAAAAACAGGCAGAATCGATGCTTGAATCTA
TGGCCAGCCGTATTGAAAATTATCCTGATATAAAGCCAGAATTGAACAACATATTAGTGAAAC
CAAACATCAAATTACCATGCTCGAAGAAGTGTTGGACCGTAATGGCATTTCCCGTTCGGTGTTG
AAAGACTCCATGAGTAAAATGGCAGCAATGGGGCAATCTATCGGTGGCATGTTCCCTTCCGATG
AAATTGTCAAAGGTTCAATTAGCGGTTATGTTTTCGAGCAGTTCGAAATTGCTTGTTATACCTC
CCTGCTGGCGGCGGCAAAAAAAGCTGGCGACACTGCCTCAATTCCGACGATTGAAGCCATTCTG
AAAGAAGAAATGCAAATGGCTGACTGGCTTATCAAACATATTCCGCAGACAACGGAACAATTTT
TACTGCGATCTGAAGCAGATGGCGTTGAAGCCAAAAAA**TAA**ataataagcaggaggcaat**ATG**T
TTCGACACGTAAAACAACTTCAATATACTGTGCGAGTGAGCGAACCTAATCCTGGATTAGCGAA
CCTGCTGCTGGAACAGTTTGGCGGCCCGCAGGGCGAACTGGCGGCCGCCTGCCGCTACTTCACG

>STM14_RS08675_*treZY*_unknownTSSs

tttgttgacctcaatccgcagacgatggcgccagtcgctttctgggtgctgaatgaagatgaag
attttaaaggcggggactacgtagatttccaggaaactgagacgacagcagtgccgctagccgt
tgagctttgtaagaaaaacccgcagagtgaattaagcaaaataaaagacgaaatcaagaaagaa
ctctcaaaataagagtaaactgatatcaaaacccggcctgtgtgtcgggttttactatttgtga
caccgtcacaaataacatcttccatttcttctaccaatcacgtggtattgcactattttcatta
gagccctttcactatatggagaccta**ATG**AAAATTTTACCGCTGGCACTCTTTATCATTCCTTT
TCTGGCCGGATGCGGCGCCAATAATACGCCGCCGCAAACACCTATTCCCGGGGAAAAAACCTCT
GCCAAATTACGTACCCTGGAAACAGGCGCGGCGGCTATTCAATCCAGACCACCTGTCGATGCCA
TCAGTACCTACCTTGACGGGTTCCATTTTTATAGCGGTGATAAAAACGGACAGATGGAAGCGCA
CCATTACGTTACCGTCCTGAACGAAGATGTCATGCAGGCGGTGATTTACGACGGCAATACGAAA
AACGCGCGCCTGATGGGGGTGGAGTACATTATCAGCGAACGTTTATTTAAAACGCTTCCTCCCG
AGGAGAAAAAACTGTGGCACAGCCACCAGTACGAGGTGAAATCCGGTAGCCTGGTGGCGCCTGG
CTTACCGCAGGTCGCTGATAAAGCGTTGATGAGTAAGATTGTTAATACTTATGGCAAGACCTGG
CACACCTGGCATACCGACCGGGATAAAACCCTGCCAATGGGTATCCCTGCGCTGATGATGGGCT
TTACTGGCGACGGGCAGCTTGACCCCGCGCTGCTGGCCGATCGGGATCGCCGTCTGGGAATTGA
TACCCAAGCCATTAAACGCGAGCGGCAGGATCTGCCTGAACATCCCGTTGTTAAGGGGGCTAAC
GCCTGGGAGCAGGGAGAGGTTATCCAGCTACAGCGTGTTCAGGGCTCTGGCGAACACGGGCGAG
GCGATACCGCGCACTTCGGTACATCTGAGCAATCCCGACAA**TAA**ttccacggttttacacgcag
atacgaaaaagaatgcctgttaagtgacagcgtcacgtaaagattagccccatagtctatgctt
gttcttcggatgttgtagaggttatgggttaaacggagcgacaa**ATG**

>*fbaB*

cgaacatcatgaacgacagtttaaaggtcgttttcattttgaatccttcatgacaggtaaggt
acgccatgacaataccatttaataatacctttaaataccattgagcattaattttgaccgatc
taacactttttcggacgtgaatcgagtcagcagatagcgccgaaaaatgcgctaaagttggca
AaaatggtcggtcagtaccaggcttattgcGacccgcaatctgcggggcaggactattcacctt
ccgataccctatcggaacttacgggagcatagct**ATG**

>*talA*

agggcgctttgttttaactgctcatccatcttgttgttcctttcacgtatcgttcaaaaagtga
cgcggcctggtgcgccgcacattatcaccgtactgcgcaacacaaactttgaaggtctgcaaac
tatcacgacgcagcgcaagagtttgttatcaacttataacggtcatgttacctgcttataccag

bioRxiv preprint doi: https://doi.org/10.1101/2023.03.23.533958; this version posted March 23, 2023. The copyright holder for this preprint (which was not certified by peer review) is the author/funder, who has granted bioRxiv a license to display the preprint in perpetuity. It is made available under aCC-BY 4.0 International license.

caataccatgcctgtctgctAtgcttttggttatgtagtgtatcaatctttcagaagtgtgaa
tcaacacactcatctaacactttacttttcaaggagtatttcct**ATG**

>*cstA*
ttgcctgccgctgcatctggggcggcagaatcaaagctgggaaattacgctctttgacgagcag
gggaggcgctgctgcacctgtcgcctgggaacggcggtaatgggatagttctggcaaagtaaaa
tgcaattgcagcattaacgaagaaaggaagtgatctggttaacactgtaatgtaaaaatgggt
Aacaatccggttttatcat**gttgttgtgttgttaattacggtaaaggtgttatagaacaaaat**
Gtaacatctctctggaacacccaaacggacaacaact**ATG**

>*ybdD*_unknownTSS (STM14_RS03610)
gcggcaatattccgccgcaatacaccgaatcgcaactcgcgcagttggtattcaataaccgtct
ggatgccgggctaaccatcttctttatggtggtggtcgtggtgctggcggtcttctctattaag
acggcgctggccgctctgaagattgataaaccgacggcgaatgaaacgccgtatgagccgatgc
cggaaaatgtg**gatgagatcgtgacgcaggcgaaaggcgcgcactaatcttttac**atcaatag
ccctctccagaataaggagaggcatttgaatgacaggtgacaacg**ATG**

>*ybdH* (STM14_RS03615)
gcgcgacatgataagccaaccgctcgtgcaaataacgaggaccatcaaaatcaggaagccttg
cgataaatttattgcctgatgtttctgcgccagcgcgctcatttgcgtaaaaatcgtggtacca
agatttggcaatttgcttttggaatcagtgggttatttctcattatcttcatacccgccagt**g**
atgtggttgttgacacactatcacagggtgttagtatttggcaAtcaagacgtttagatgtcta
aatataataacaacggtgagaagaccctaaggacaacacaac**ATG**

>*ybdR* (ST14_RS03680)
acccaggcagtacatgcaacagatgaatgacgccatcctgttgcgccagaaactcagcgtggcg
aatggctttatcgctaagttccatttcaaaaacatcaaccggcataatgattgtcttatacata
cccgttac**tccctgttaatcaatactgaatttattcaagcatataataatgtt**Tctttc**tgcta**
tttgtctggcaattttaaatattacggcaacctgatgttaaaaaagttgtgtatttccaggaa
aaatcctaaagccgcctgttttgccggcagtctctccgggatagctaccctttatagtcgaac
gtgacattcaccgttaccccggaggctgc**ATG**

>*wrbA*
tgacctccgtcatagattagacatcatattgcataggtaatgaggagtatctcctcgcgcagcg
tatgaacgatatcatcagtaacgccgcgtaacattaagcatagtgaaaaacgtcgtagtaggcc
gcttaccgaaatattctgcaacattggaagccgaatacgcagaataataaccataagaaataaa
taagttattctt**tatttgggaaaatcaaagcgcttaaaaatgtatcattttgcaa**caaaggtc
gccaaattaattattgttataaatcaaagaaatg**gcgctgaaatttgcactcttcacaggagag**
catatctttaaataGacgtagcgaatcgctacacacctgattaatctgaggagtagtacaa**ATG**

>STM14_RS08755_unknownTSS
tgtactggggattccggaaatggccgatcccgcgtttaaattccatctggatcgta**tgattacg**
cttgaactctttgcgtaagttacaaataatttaataaaaatcgcttttaatttacaaatagta
acaattaagaaacatcatatctcctcatcgcgccgcgctggcctggtttttatctattctttc
actcaatattctaaaaaacttcaataagttctcaaaagagcgcgtgacgctctgtctctttt
tgtgttttcttcaaatgatatgcgcatgtgaggggtaaa**ATG**

>*yohF* (STM14_RS12065)
gccagtcaggtagagacgctggcgcagaaaaatcagcatcagcagcagattgagcatgacgcgc
agcgtgt**ggtcggtctggcgcaagcgcgctttaatgccggtattattgcggg**ttcccgcgtgag
cgaggccaaaatcccggcactgcgcgagcagtgtaacggcttgctgttgcagggccagtggctg
gatgcctctattcagcttaccagcgcgctgggcggcggttatcattc**ctgatttttccggatga**
ttcagacagggttcctatacttaggctGtgtatgattcatccggagaggaat**ATG**

>*lysM*(STM14_RS15000)
ctcttttaaacaatgactaattttgcttgtatggagtgtatatgggtttctggagaattgtat
ttacgattattttgcctccgctcggcgtactttaggaaaagggttcggttgggcatttatcct
taatatcctgctgacattactcggatatattccggggcttatccacgcatttgggttcagatg

bioRxiv preprint doi: https://doi.org/10.1101/2023.03.23.533958; this version posted March 23, 2023. The copyright holder for this preprint (which was not certified by peer review) is the author/funder, who has granted bioRxiv a license to display the preprint in perpetuity. It is made available under aCC-BY 4.0 International license.

cgtcattaattaccggccaccggcggcgcataagcgccgcttatcaggataacctcattccctc
tgctacacttttctttGtgtgtatttttgacaagcgaggtgaat**ATG**

>*apeE*_unknownSTT (STM14_RS03455)

cgggaactggcgctggcacgttttcgaaagcgcgcagtactatccagtgcaaaataatatgca
cgacaactgctgtcagaaaaataataccaaatatcaccaccagcgacgtggtgtgattcatttc
aattcccgctaaatcttctacccgggatatcaattcctgcataatctctcctttataaacagca
gcctatgatgactgctgaatggcatttatgcaaatcaggacgcctgcgcttcaggcataatcag
caacagacgggtaaaagcgcctctggggttatccatacgctatattttatcctgcgccctgtaa
acgtcgttattttacgcggttccctcttttgaacaggcgccagtccgtcaacaatgaatgaaa
actttttaaccagaatgtttaaaacgattgcaatatctctgtcggataatgattagcaccgtcg
tacctatcaacaataacattaaggaacatcgg**ATG**

>*yciG* (STM14_RS09540)

acgtcaatttcccccgcctttatgtaaagaaatgtcaacaaacaatgcattaacgtttccaggg
ccgcagatagtgacgattgttattaataacaatgcaaagcatttcctgttaaatcgcttattct
aaaagaaaatatagaatatattatctctaaaccatttttgtgcgttatttacgcggtaaatca
caactatttccgtcaatttgactaatcggtttaaccaactaattttaataggGtgtcgacacgg
ttaaaaccgatttttcagcaagcaacgagacaggagaaataat**ATG**

>*bapA* (STM14_RS14685)

gaagctggtcaatgttcatatctcatgcatgcctcgaaaatatattctctgtgtctgacgctta
tcaatagcaataaataccagaacaagaatatattctttgtttaaaaatttttatagtctaataa
cacaatacttcaaatgatttttataaaatttaatattgctgaataccttcacaaaaagtattgt
ttttatgttaaaagagaaaaagaagaaaaattaaaattcattataaaacataaagataaataa
aaaattatcatgtattgttagcccaccctacatctccttaacacaaactccgttatatttcaga
cgcactaacacctttatcaatagatcttaatttgcagaaagatttttttctgactatatcatct
aatAcgaaagcactagtcaggcacaaaaaacaaagggttattcggcgagaaaaccagacctcca
cctacgctcataaaaaagaatatggctacggaaattcatctctcacgatgaacgggaaggctcg
tctacgcatttttgccctgaacgttgtgcccgttaattaatacacagagcaaatccatcaggagc
tgattt**ATG**

>*phoN2* (STM14_RS19030)

actgataacgccccgcgcccgcgtgcgtgtccagatagagaaacggtttttctttctcttttag
cgactcgatgatcaggctctgaacggtatgtttaaggacgtcggcgtggttgccagcgtgaaag
ctgtgacgataactgagcatggatgcagatattccgggaagtaaacaagttagccgatagttta
ccgcagatccgtagagattaccccagtaccgccatgttgtttcgcgcgttgtgaaagtattgca
agacgtgttcgtatcatcaaaccGtcaactgcgcaggccagcatcgcctgtgggcaaggtagta
gttttgacctggcgttattttttaatgaaaggaaccata**ATG**

>STM14_RS08420

aacttctgatgacagccagttaaatttatggggttaagttgtctgaatataaagagggcgtgca
ttcggatttttctacttatttttccgtggtggctcgattgccaaagatttattaccgcttcgct
gttgatgttttcaaaaaatatatcttccgccatgtgatggtattatgtaaataatgcagaaatg
aatttgacactgcgcacagggcgactagatttagaactGtatcacatgatatgagaagacatat
catatttaaaacgcaacatcatcatgaggattatatt**ATG**

>*ymgE* (STM14_RS09875)

caaaatttactgatgaattgaccatgcgcccatgaaatacgcagtggaacctgatttttattt
aaatcccgtagcacgcctaatatcgctaatggatttttttttcaggaactgctcattgtaagcac
tcacacgaaataacccctgatacgcagactatcgttgggttatcggcatcggtagaaagaactt
aatagcgataaataaattttatgtccgcgagataaatcagtgttcagaaatgcgacgctcgac
tttgcctatacttaaaacGtttttactggaaagagggttcgcaa**ATG**

>STM14_RS19935

tcgcctccgccgtaaacgccgtctcctgcgccaaatcggcaagacatagcccgcgcgtcagccg
ttccagctctcccagtacgatctcaatacgtctcattacactaccgcttacgaaataaaagagt
agtcagaacatacactattctgccacgcaagaaagtgaaggcgcagcgagaaaccgccttccca

bioRxiv preprint doi: https://doi.org/10.1101/2023.03.23.533958; this version posted March 23, 2023. The copyright holder for this preprint (which was not certified by peer review) is the author/funder, who has granted bioRxiv a license to display the preprint in perpetuity. It is made available under aCC-BY 4.0 International license.

cttttgcctgacttcaacgccaacggata**gcacgccccgatgagtgaacgtcgcacggtctaca**
cttactctt**G**aaaaagtgcaaaccgataaggataccgttt**AATG**

>*STnc4130* (sRNA at 3′-end of STM14_RS23700)

aaaccag**aaaaatacattgagaaaaagattaatgatattaatataaatgat A**taaaatagtgag
cacgcataacataagggcatagtaatatatcataaaatcctttaattactattgcccttgaata
gattcactattccacatccttcctttccaacccgatatcccttaactgctcgtcactcattcgt
cgcagcgcctgacaggcccgcttacgcagccaccaccttttttagcgcccgtctgagctgcacaa
agccaatga**ATG**

> STM14_RS23900_unknownSTT

gccaagttgctggcggacgatctcgtcccttcccgtaaagtgaaagtggaaacgaccgatggcg
tcgtacagctctccggtaccgttgaaactcaggaacaaagcgaccgcgctgaaagcatcgcgaa
agccgttgatggcgtaaaaagtgttaaaaacgatctgaaagttcagtaattcgtcgtaattcgt
c**ctcccgacgtttgtcgggaggcgtaatgtgcaccacactaaaaatG**tcgcgaatgagtagcct
gagcgctcatatttagcggtcgacattaactatggtaaaggagaggctt**ATG**

bioRxiv preprint doi: https://doi.org/10.1101/2023.03.23.533958; this version posted March 23, 2023. The copyright holder for this preprint (which was not certified by peer review) is the author/funder, who has granted bioRxiv a license to display the preprint in perpetuity. It is made available under aCC-BY 4.0 International license.

Supporting Figures

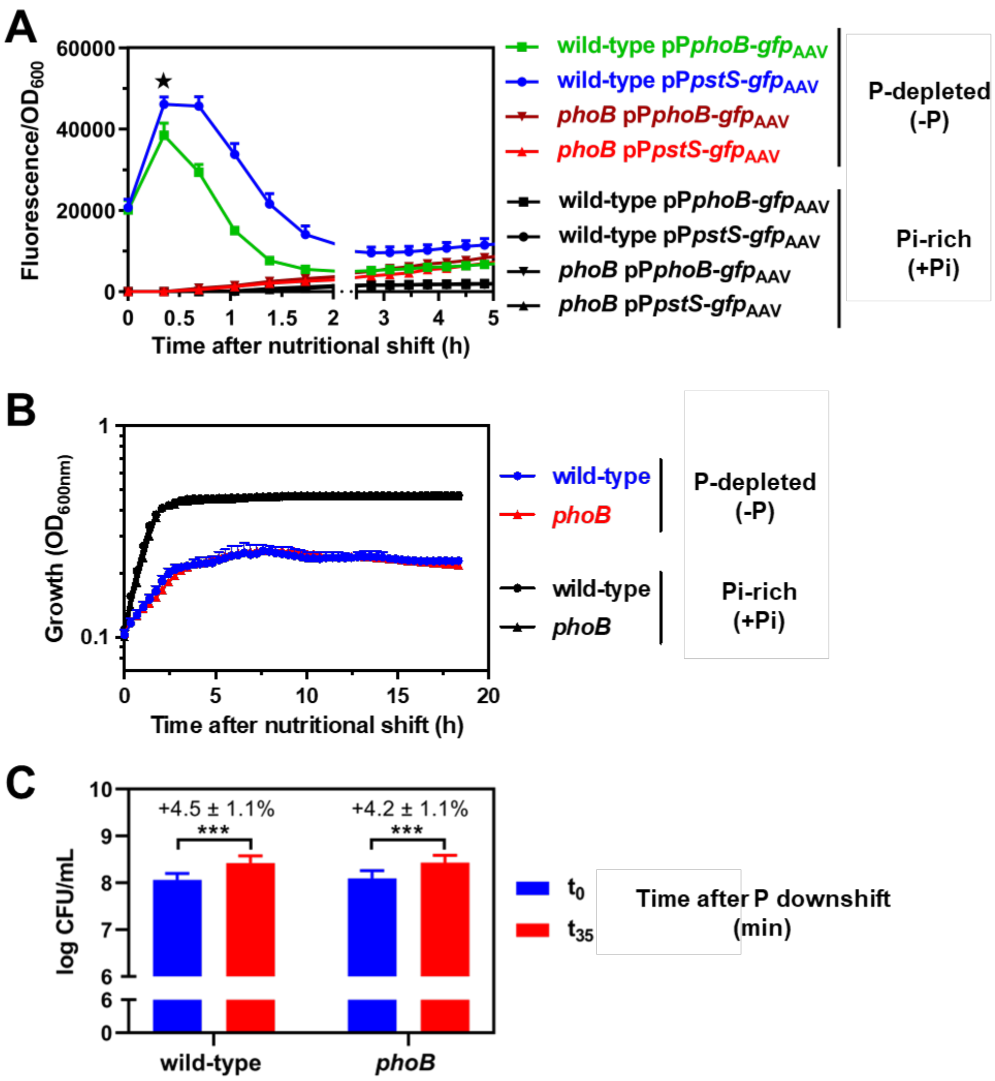

Figure S1. (A) Fluorescence from wild-type (14028s) and *phoB* (EG9054) *Salmonella* carrying pP*phoB-gfp*AAV or pP*pstS-gfp*AAV. Cultures were propagated to mid-logarithmic phase in MOPS medium containing 1 mM K_2HPO_4. Subsequently, cultures were either subjected to a nutritional downshift to MOPS medium lacking K_2HPO_4 or any other alternative P source (-P) or maintained in the same medium containing 1 mM K_2HPO_4 (+Pi) as control (see Materials and Methods for further experimental details). The maximum GFP expression from *phoB* and *pstS* transcriptional fusions occurs at 35 min following the removal of P, and is indicated with a ★. **(B)** Growth curve of wild-type (14028s) or *phoB* (EG9054) *Salmonella* harboring pP*pstS-gfp*AAV subjected to the same nutritional shifts described in (A). **(C)** Viable cell counts of wild-type (14028s) and *phoB* (EG9054) *Salmonella* at the beginning (t_0) and the end (t_{35}) of Pi downshift. *** $P < 0.001$, paired two-tailed *t* test. In all cases, means ± SDs of at least three independent experiments are shown.

bioRxiv preprint doi: https://doi.org/10.1101/2023.03.23.533958; this version posted March 23, 2023. The copyright holder for this preprint (which was not certified by peer review) is the author/funder, who has granted bioRxiv a license to display the preprint in perpetuity. It is made available under aCC-BY 4.0 International license.

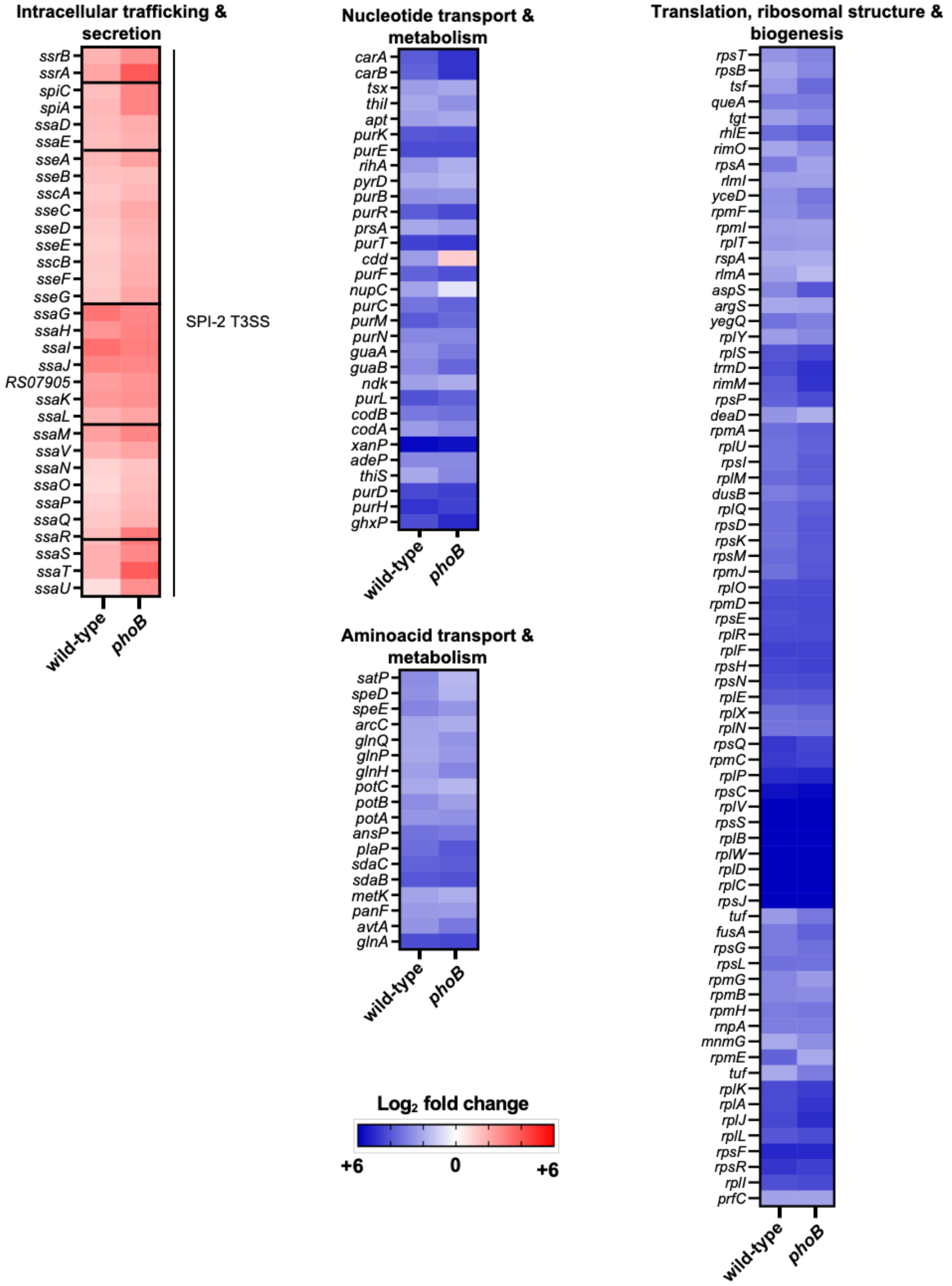

Figure S2. *Salmonella* PhoB-independent response to P starvation. Heatmaps depicting fold changes in transcript levels between -P and +Pi treatments for wild-type (14028s) and *phoB* (EG9054) *Salmonella*. Graphs show selected transcripts from genes that show no significant changes between wild-type and *phoB* during P starvation (Table S1). Displayed genes are organized by COG categories. Note that gene *RS07905* corresponds to NCBI gene locus tag *STM14_RS07905*).

bioRxiv preprint doi: https://doi.org/10.1101/2023.03.23.533958; this version posted March 23, 2023. The copyright holder for this preprint (which was not certified by peer review) is the author/funder, who has granted bioRxiv a license to display the preprint in perpetuity. It is made available under aCC-BY 4.0 International license.

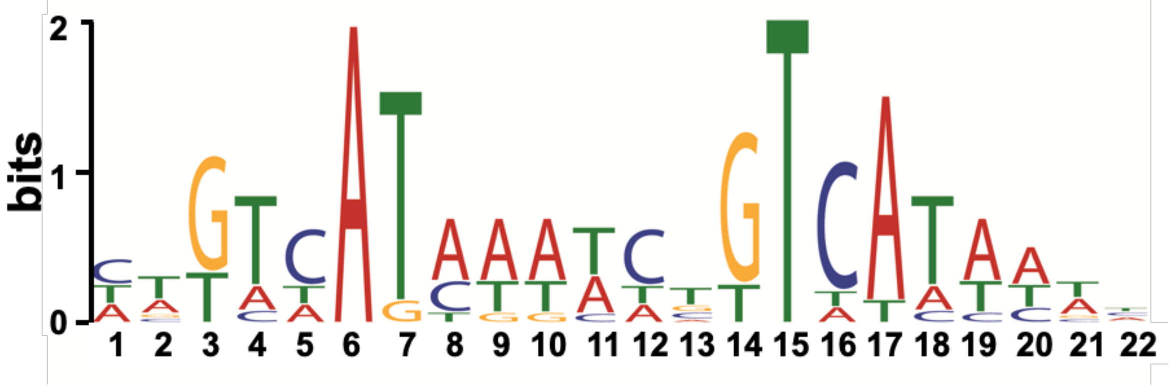

Figure S3. ***Salmonella*** **PhoB binding motif.** Sequence logo of the PhoB binding sites. The logo was generated by the MEME algorithm using as input 450-bp upstream of the translation start site of the canonical PhoB-activated operons listed in Table S3 (6).

bioRxiv preprint doi: https://doi.org/10.1101/2023.03.23.533958; this version posted March 23, 2023. The copyright holder for this preprint (which was not certified by peer review) is the author/funder, who has granted bioRxiv a license to display the preprint in perpetuity. It is made available under aCC-BY 4.0 International license.

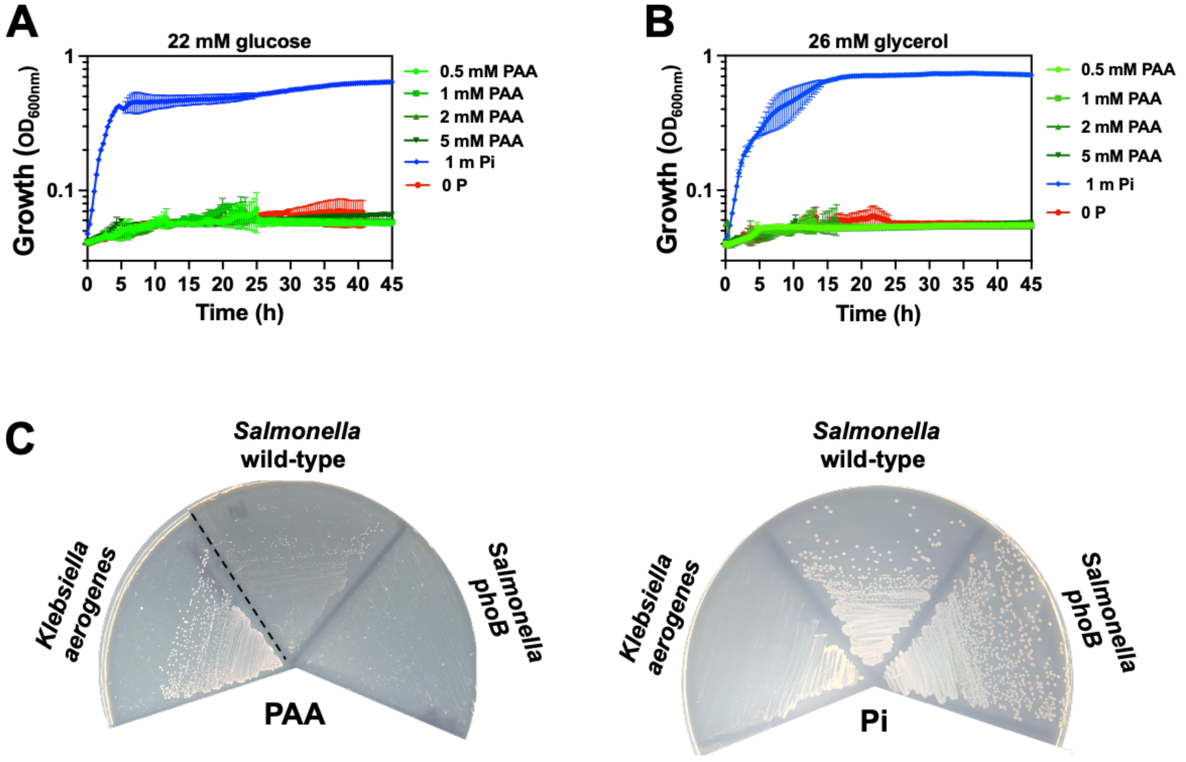

Figure S4. Utilization of phosphonoacetic acid as sole P source. Growth of wild-type *Salmonella* (14028s) in MOPS liquid medium containing (**A**) 22 mM glucose or (**B**) 26 mM glycerol as carbon source and either no P, 1 mM Pi (K_2HPO_4) or the indicated concentration of phosphonoacetic acid (PAA) and sole P source. Growth curves show the means ± SDs of three independent biological replicates and are representative of two independent experiments. (**C**) Growth of wild-type *Klebisiella aerogenes* (ATCC13048), and wild-type (14028s) and *phoB* (EG9054) strains of *Salmonella* on MOPS-glucose-noble agar plate containing 1 mM PAA (left) or 1 mM Pi (right) as the sole P source. Plates were incubated at 37°C during 14-18 h before being imaged. Images are representative of three independent experiments. Dashed lines separate non-contiguous sections of the same plate. Note the presence of P-compound(s) in the noble agar that allows the formation of small *Salmonella* colonies. This is not observed during growth in liquid medium (A).

bioRxiv preprint doi: https://doi.org/10.1101/2023.03.23.533958; this version posted March 23, 2023. The copyright holder for this preprint (which was not certified by peer review) is the author/funder, who has granted bioRxiv a license to display the preprint in perpetuity. It is made available under aCC-BY 4.0 International license.

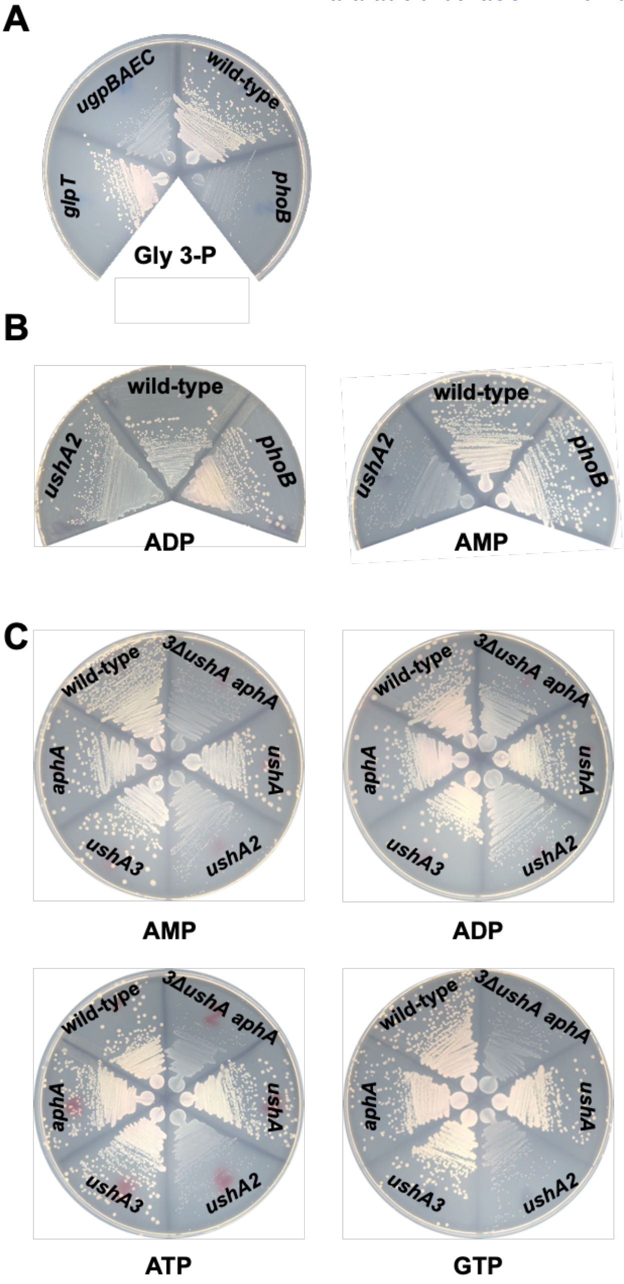

Figure S5. *Salmonella* genetic requirements for the utilization of glycerol-3-phosphate and nucleotides as the only P source. (A) Growth of wild-type, *glpT* (MP1735), *ugpBAEC* (MP1736) and *phoB* (EG9054) *Salmonella* strains on MOPS-glucose-noble agar plate containing 0.5 mM of *sn*-glycerol-3-phosphate (Gly 3-P) as the sole P source. **(B)** Growth of wild-type, *ushA2* (MP1779), and *phoB* (EG9054) *Salmonella* on MOPS-glucose-noble agar plates containing 0.5 mM of ADP or AMP as sole P source. **(C)** Growth of wild-type, *ushA* (MP1778), *ushA2* (MP1779), *ushA3* (MP1780), *aphA* (MP1785) and *ushA ushA2 ushA3 aphA* (*3ΔushA aphA*; MP1796) *Salmonella* strains on MOPS-glucose-noble agar plates containing 0.5 mM of either adenosine monophosphate (AMP), adenosine diphosphate (ADP), adenosine triphosphate (ATP) or guanosine triphosphate (GTP) as sole P source. In all experiments, plates were incubated at 37°C during 16-18 h. Images are representative of two independent experiments.

bioRxiv preprint doi: https://doi.org/10.1101/2023.03.23.533958; this version posted March 23, 2023. The copyright holder for this preprint (which was not certified by peer review) is the author/funder, who has granted bioRxiv a license to display the preprint in perpetuity. It is made available under aCC-BY 4.0 International license.

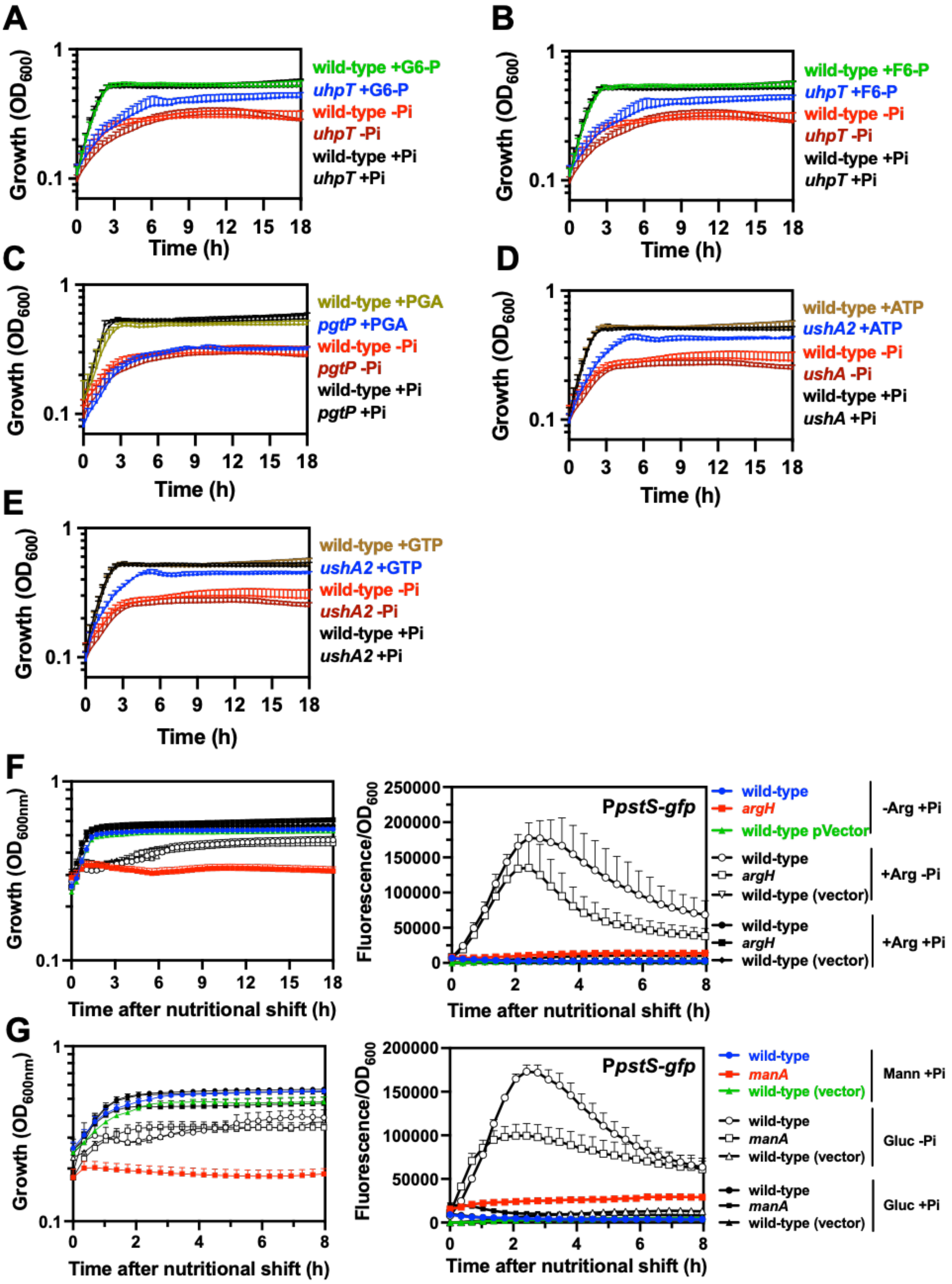

Figure S6. Effect of growth rate on the activity of the P*pstS-gfp* transcriptional fusion. (A-E) Growth from wild-type (14028s), *uhpT* (MP1738), *pgtP* (MP1739) and *ushA2* (MP1779) *Salmonella* carrying pP*pstS-gfp*. Growth curves are derived from the experiments outlined in Fig. 6. Prior to performing the readings, cultures were grown to mid-logarithmic phase in MOPS medium containing 1 mM Pi (K_2HPO_4), washed in MOPS medium lacking a P source and resuspended in MOPS medium containing 1 mM of the indicated organic P source (G6-P: glucose 6-P; F6-P: fructose 6-P; PGA: 3-phosphoglyceric acid; ATP: adenosine triphosphate), 1 mM Pi (K_2HPO_4) or lacking P. Means ± SDs of at least three independent experiments are shown. **(F)** (Left) growth curve

bioRxiv preprint doi: https://doi.org/10.1101/2023.03.23.533958; this version posted March 23, 2023. The copyright holder for this preprint (which was not certified by peer review) is the author/funder, who has granted bioRxiv a license to display the preprint in perpetuity. It is made available under aCC-BY 4.0 International license.

and (right) corresponding fluorescence of wild-type (14028s) and *argH* (EG6537) *Salmonella* harboring pP*pstS-gfp* or the vector control (pVector, pFPV25). Cultures were grown to mid-logarithmic phase in MOPS medium containing 1.6 mM arginine and 1 mM Pi (K_2HPO_4), washed in MOPS medium lacking arginine and a P source. Cells were subsequently resuspended in either MOPS medium containing or lacking 1.6 mM arginine (+Arg; -Arg) and 1 mM K_2HPO_4 (+Pi; -Pi). Growth and green fluorescence were then monitored for 8 h. **(G)** (Left) growth curve and (right) corresponding fluorescence of wild-type (14028s) and *manA* (MP50) *Salmonella* harboring pP*pstS-gfp* or the vector control (pVector, pFPV25). Cultures were grown to mid-logarithmic phase in MOPS medium containing 22 mM glucose and 1 mM K_2HPO_4. Cells were washed in MOPS medium lacking carbon and P and then resuspended in MOPS medium containing either 22 mM mannose (+Man) or 22 mM glucose (+Glc) as C sources, and either with or without 1 mM K_2HPO_4 (+Pi; -Pi). Growth and green fluorescence were monitored for the next 8 h. Graphs depicted in F-G show the means ± SDs of at least three biological replicates are shown and are representatives of at least 3 experiments.

bioRxiv preprint doi: https://doi.org/10.1101/2023.03.23.533958; this version posted March 23, 2023. The copyright holder for this preprint (which was not certified by peer review) is the author/funder, who has granted bioRxiv a license to display the preprint in perpetuity. It is made available under aCC-BY 4.0 International license.

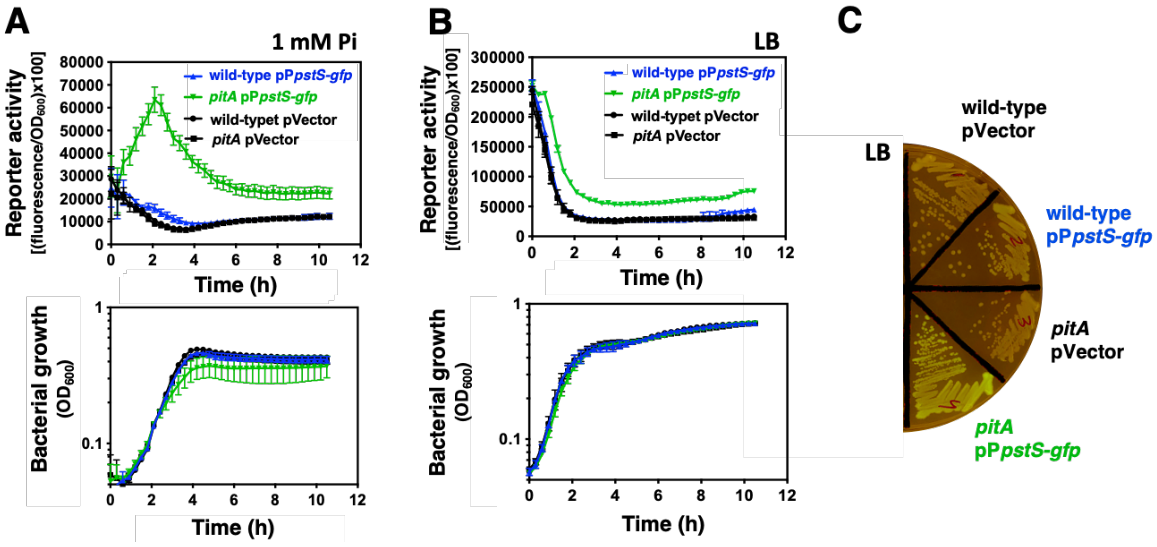

Figure S7. Effect of *pitA* deletion on the activity of the P*pstS-gfp* transcriptional fusion. (A) (Top) Fluorescence and (bottom) growth of wild-type (14028s) and *pitA* (MP1251) strains of *Salmonella* harboring pP*pstS-gfp* or the vector control (pVector, pFPV25). Measurements were performed during growth in MOPS glucose medium supplemented with 1 mM Pi (K_2HPO_4). **(B)** (Top) Fluorescence and (bottom) growth of strains described in (A). Measurements were performed in LB medium, which contains 2 mM Pi (81). **(C)** Fluorescence of strains described in (A) on LB plates. Plates were incubated at 30°C for 14-16 h. Image is representative of three independent experiments.

bioRxiv preprint doi: https://doi.org/10.1101/2023.03.23.533958; this version posted March 23, 2023. The copyright holder for this preprint (which was not certified by peer review) is the author/funder, who has granted bioRxiv a license to display the preprint in perpetuity. It is made available under aCC-BY 4.0 International license.

Supporting Information References

1. Fields PI, Swanson R V., Haidaris CG, Heffron F. Mutants of *Salmonella typhimurium* that cannot survive within the macrophage are avirulent. Proceedings of the National Academy of Sciences. 1986;83(14):5189-5193.
2. Pontes MH, Groisman EA. Protein synthesis controls phosphate homeostasis. Genes and Development. 2018;32(1):79-92.
3. Bruna RE, Kendra CG, Groisman EA, Pontes MH. Limitation of phosphate assimilation maintains cytoplasmic magnesium homeostasis. Proceedings of the National Academy of Sciences. 2021;118(11):e2021370118.
4. Pontes MH, Groisman EA. Slow growth determines nonheritable antibiotic resistance in *Salmonella enterica*. Science Signalling. 2019;12(592):eaax3938.
5. Datta S, Costantino N, Court DL. A set of recombineering plasmids for gram-negative bacteria. Gene. 2006;379:109-115.
6. Datsenko KA, Wanner BL. One-step inactivation of chromosomal genes in *Escherichia coli* K-12 using PCR products. Proceedings of the National Academy of Sciences. 2000;97(12):6640-6645.
7. Lee TS, Krupa RA, Zhang F, Hajimorad M, Holtz WJ, Prasad N, et al. BglBrick vectors and datasheets: A synthetic biology platform for gene expression. Journal of Biological Engineering. 2011;5:12.
8. Valdivia RH, Falkow S. Bacterial genetics by flow cytometry: rapid isolation of *Salmonella typhimurium* acid-inducible promoters by differential fluorescence induction. Molecular Microbiology. 1996;22(2):367-378.
9. Soncini FC, García Véscovi E, Groisman EA. Transcriptional autoregulation of the *Salmonella typhimurium phoPQ* operon. Journal of Bacteriology. 1995;177(15):4364-4371.
10. Coppens L, Lavigne R. SAPPHIRE: a neural network based classifier for σ70 promoter prediction in *Pseudomonas*. BMC Bioinformatics. 2020;21(1):415.
11. Bailey TL, Johnson J, Grant CE, Noble WS. The MEME Suite. Nucleic Acids Research. 2015;43(W1):W39-W49.
12. Kröger C, Colgan A, Srikumar S, Händler K, Sivasankaran SK, Hammarlöf DL, Canals R, Grissom JE, Conway T, Hokamp K, Hinton JCD. An infection-relevant transcriptomic compendium for *Salmonella enterica* serovar Typhimurium. Cell Host and Microbe. 2013;14(6):683-695.
13. Srikumar S, Kröger C, Hébrard M, Colgan A, Owen S V, Sivasankaran SK, Cameron ADS, Hokamp K, Hinton JCD. RNA-seq brings new insights to the intra-macrophage transcriptome of *Salmonella* Typhimurium. PLoS Pathogens. 2015;11(11):e1005262.
14. Jarvik T, Smillie C, Groisman EA, Ochman H. Short-term signatures of evolutionary change in the *Salmonella enterica* serovar typhimurium 14028 genome. Journal of Bacteriology. 2010;192(2):560-567.
